# Psychopathological Symptoms and Personality Traits as Predictors of Problematic Smartphone Use in Different Age Groups

**DOI:** 10.3390/bs12020020

**Published:** 2022-01-25

**Authors:** Lea-Christin Wickord, Claudia Quaiser-Pohl

**Affiliations:** Institute of Psychology, University Koblenz-Landau, 56070 Koblenz, Germany; quaiser@uni-koblenz.de

**Keywords:** problematic smartphone use, psychopathological symptoms, personality, big five, generations, development

## Abstract

The study investigates psychopathological symptoms and the Big Five personality traits as predictors of “problematic smartphone use” (PSU) in different generational groups. The generational groups were selected to analyze whether the different life stages and developmental tasks that need to be completed have an impact on PSU. The groups were divided into digital immigrants, digital natives, and Generation Y and Z as subgroups of digital natives. A total of 399 subjects participated (312 women, 86 men, 1 diverse; mean age = 25.9; range 14–67; 44 digital immigrants, 355 digital natives, 35 Generation Y, and 320 Generation Z). They completed the ICD-10 Symptom Rating (ISR), the Big Five Inventory-10 (BFI-10), and the Mobile Phone Problematic Use Scale (MPPUS). The results show differences between digital immigrants and digital natives in the expression of PSU, neuroticism, conscientiousness, depression, anxiety, and compulsivity. Concerning Generations Y and Z, differences were only found in the expression of the PSU. Regression revealed that symptoms for obsessive-compulsive disorders, depression, conscientiousness, and gender were significant predictors of PSU. Moderations have shown that being a digital immigrant/native moderates the impact of eating disorders on PSU. Belonging to Generation Y/Z moderates the influence of conscientiousness and depression on PSU. Thus, it shows that in different generations, different factors seem to play a role in the development and maintenance of PSU.

## 1. Introduction

Smartphones are considered among the most widespread and popular communication devices [1,2]. In 2019, around 81.7% of Germans over the age of 14 owned a smartphone [3]. In this context, it can be seen that the smartphone medium has shaped and changed the existing communication and information landscape enormously. Information and contacts are now easier to access, faster to find, and spread over a larger radius [4,5]. Most needs of smartphone users can be satisfied with the help of their smartphones [1]. Smartphones facilitate a variety of life domains, providing, for example, sociability, entertainment, distraction, information gathering, time management, coping strategies, and enacting one’s identity [6,7,8,9,10]. Thus, the handy telecommunication device becomes an indispensable part of everyday life [11].

### 1.1. Problematic Smartphone Use

Alongside these positive results, concerns about user overuse and dependency are growing louder. For example, several studies have already found that some users are so influenced by their smartphones that they sense separation anxiety when they do not have the device with them [12,13]. This phenomenon can be found under the term problematic smartphone use, where excessive smartphone use is assumed, and symptoms similar to substance-related addictions, functional impairment, and withdrawal are evident [14,15]. Billieux refers to problematic smartphone use (PSU) as “an inability to regulate one’s use of the smartphone, which eventually involves negative consequences in daily life.” [16] (p. 1). Due to the similarity of symptoms to addicts, such as loss of control, mood regulation, or loss of cognitive functioning, the term “smartphone addiction” can be found in many studies [17,18]. Smartphone addiction itself refers to an addiction that disrupts users’ social connections through excessive and unregulated usage [19]. As Kuyulu and Beltekin point out: “The use of smartphones both gives pleasure to the person as a result of use and saves them from pressure or anxiety. Such reinforcement makes it easy to be addicted to the smartphone” [1] (p. 305). Despite these addiction-like symptoms, it is important to distinguish problematic smartphone use from addiction according to ICD-10 criteria, as the consequences of this do not resemble the intensity and severity of addiction consequences [11]. To avoid pathologizing smartphone use, Long et al. suggest using the term “problematic smartphone use” (PSU) for the phenomenon [20]. This term is also used hereafter. Although the similarity of symptoms does not entirely meet the necessary criteria of addiction, studies showed that PSU could have negative impacts on fatigue, insomnia, diminished immune system, wrist syndrome, neck muscle fatigue, social isolation, family problems, neglect of daily chores, cyberbullying, sexual assault, and school failure [20,21].

When considering PSU, it is crucial to take a more nuanced look at the smartphone because it is not the technological device itself that is addictive, but the range of uses that the smartphone offers, which serve to satisfy needs and which, if misused, make it a space for multiple dependencies [22,23,24,25]. In this sense, for example, the need for connection can be liberated by social media platforms, which, however, can have negative consequences for one’s emotions, relationships, psychological stability and can even lead to depressive symptoms if consumed excessively [26,27,28]. In addition, Markowetz determined that the constant digital alertness overtaxes our cognitive, psychological, and social abilities [29]. For these reasons, more and more mass media are covering the gravity of the problem, and studies of the symptoms of overdependence, as well as the development of appropriate measurement tools, are steadily increasing to predict fewer health issues regarding the usage of smartphones [16,30].

### 1.2. Mental Health and Psychopathological Symptoms

Mental health, in particular, plays a significant role when considering PSU. The World Health Organization (WHO) defines mental health as a state of well-being in which the individual is able to use his or her faculties, cope with the regular life stresses, and give back to his or her community and has acknowledged PSU officially as a public health concern [31]. Digitalization and constant accessibility for one’s own community, in particular, ensure a “fragmentation of everyday life” and possibly lead to overload [29]. Looking at the current state of research on psychological stress factors in relation to the digitization and the use of new technologies, it is clear that information and communication technologies, in particular, are associated with a range of psychosomatic complaints, signs of stress, exhaustion, and depression [32,33]. These include symptoms such as sleep disturbances, headaches, persistent fatigue, and risky sexual or aggressive behaviors [28,31,34,35,36]. For example, Sampasa-Kanyinga and Lewis examined the association of time spent on social networking websites and psychological functioning in children and adolescents [37]. They found that poor mental health (self-reported), high levels of psychological overload, harmful stress, and suicidal thoughts were independently associated with spending much time on social media. Moreover, Lee et al. showed that increasing the frequent usage as well as the length of the use was strongly correlated with the gravity of depression and depression severity correlate with PSU [38,39,40]. Similar correlations, only less pronounced, are also shown for anxiety and its severity [41,42,43,44]. The findings of Kim, Seo et al. show that smartphone use to reduce negative emotions mediates the association of depression and anxiety severity and PSU [45]. Subsequently, Elhai et al. revealed that behavioral activation mediates the correlation between depression and PSU [42]. Authors Kempf et al. found initial evidence of a difference in OCD according to the duration of smartphone use [46]. A correlation between PSU and OCD has been shown several times before [47,48,49]. Research has also found multiple correlations with PSU for somatization [47,49,50]. Tayhan Karthal et al. were able to show that PSU correlates with obesity and, similarly, that social media use as a part of PSU correlates with eating disorders [51,52,53]. Due to these correlations, a greater understanding of the reasons for maladaptive and problematic smartphone usage is required to prevent psychological health issues [14]. Most of these studies show correlations between psychopathological symptoms and PSUs without specifying a direction of action. However, some studies have also shown that psychopathological symptoms are predictors of PSU, as they lead to increased consumption of the medium [30,47,54]. For this reason, this study will also focus on the use of symptoms as predictors. The development of mental health issues is multifaceted and cannot be generalized. However, the complexity and the multitude of influencing factors, among them especially the personality of the individuals, represents a significant factor.

### 1.3. Personality and PSU

The increase in general psychological distress and the increase in smartphone use can be related and analyzed in the context of personality [55,56]. Personality is defined as the set of characteristics of an individual that determine how we perceive our environment and communicate with it [57]. One of the best-known personality models is the Big Five personality model with the five dimensions neuroticism or emotional instability, extraversion, openness to experience, agreeableness, and conscientiousness [58]. The relationship between personality and PSU has already been widely researched to determine whether specific personality traits exist that predispose smartphone users to adopt addictive tendencies or problematic usage patterns [59]. Billieux was the first to identify individual risk factors and mental characteristics associated with PSU, including personality traits [16]. The Big Five personality traits [60] have been examined in many studies on PSU [61,62,63,64]. It has been consistently found that PSU is associated with high levels of the dimensions of neuroticism and extraversion [65,66,67,68,69]. Furthermore, a relationship between low expressions on the conscientiousness and agreeableness dimensions and PSU has been demonstrated [14,59,61,64,70], and it was also shown that openness negatively predicted or correlated with PSU [63,71,72].

### 1.4. The Generational Perspective

In addition to personality, another factor that can be cited to examine smartphone exposure is age or the generation you grew up in. Most studies focus on adolescents and young adults who belong to the generation of digital natives when investigating PSU. Digital natives are defined as the young generation born after 1980 who have grown up with various IT systems from birth. Accordingly, they find digital devices easy to use and are open to technical innovations [73]. Since they are still considered the main consumers, these groups offer important clues for investigating PSU. Nevertheless, it is just as important to include other generations in the focus of the studies, primarily the so-called digital immigrants, i.e., the generation of parents of digital natives and older generations who were confronted with digital tools such as smartphones later in life, to investigate how these generations suffer from PSU [74]. While digital natives take smartphones for granted, many digital immigrants take a more critical view of constant usage behavior. It is therefore not surprising that the two groups also cite different reasons for PSU. While digital immigrants believe that PSU arises due to a lack of control by the individual user and is thus due to intrinsic characteristics, digital natives see the smartphone as the main communication tool at the center of their lives and therefore see PSU as an unavoidable evil that has arisen extrinsically due to the environment created by the smartphone (ibid).

In addition to exploring digital natives and digital immigrants, it is also necessary to look at the subgroups within digital natives, more specifically, Generations Y and Z. Depending on the definition, Generation Y includes those born between 1980 and 1996, while Generation Z includes those born between 1997 and 2010 [75]. While Generation Y, which is now 25 and older, is starting a more settled life due to age with more constant living circumstances such as a steady job, a long steady relationship, and slowly making fewer new friends [76], Generation Z is currently in early adolescence to young adulthood and has essential developmental steps to complete towards an independent life [77]. Because of these differences in developmental tasks, it is essential to examine how these subgroups suffer from PSU. Research has shown that younger individuals are more likely to develop PSU [78]. Cerniglia et al. describe that adolescents are more prone to PSU and reason that the cerebral cortex is not sufficiently developed as well as there may be a mismatch between the development of different brain areas, which can lead to both affective and behavioral dysregulation [79]. Therefore, it is important to differentiate the behavior between these two subgroups.

### 1.5. Underlying Model

Theoretically, the study presented here refers to the Pathway Model [14]. According to the authors, various pathways exist that lead to or are associated with problematic smartphone use: the reassurance-seeking pathway involves regularly checking notifications to gain social reassurance through contacts. People with lower self-esteem, higher anxiety, loneliness, and depression are associated with the reassurance-seeking pathway. The fear of missing out pathway (FoMO; the fear of missing out) is also related to smartphone checking and notifications. Other pathways in the model include the extraversion pathway, which includes symptoms of social dependence through which individuals regularly seek to establish new relationships and contacts, and the impulsivity pathway, which is characterized by a lack of self-control and includes antisocial personality traits, disinhibition, and attention deficits. As can be seen from the model, it refers both to personality factors and psychopathological aspects, which is why these two factors will be the main components of the study.

The generational approach is used because digital development has progressed immensely in recent years and thus represents a generation-shaping consideration. While older generations have only gradually integrated emerging technologies into their lives, younger generations have grown up with these devices from the beginning, leading to a changed attitude towards these gadgets [73,74]. For this reason, generational work looks at how these aspects change over the course of a lifetime. Generational cohorts can make visible how specific formative experiences (changes regarding the world, economical, technological, and social changes) affect the lives and views of individuals [75].

Therefore, the article shows the factors influencing personality and psychopathology on problematic smartphone use. For the first time, the differences in the predictors of smartphone use between generations and the corresponding developmental stages in which the individuals in the respective age groups find themselves are examined. Thus, beyond the existing research, the article offers a significant contribution to the explanation of predictors of problematic smartphone use.

### 1.6. Hypotheses

As previous studies have already shown [73,74], the smartphone is perceived differently by digital natives and digital immigrants. It is hypothesized that there are differences between digital immigrants and digital natives in the expression of PSU (H1).

Additionally, previous studies have shown that younger individuals in particular develop PSU [79]. For this reason, the subgroups of digital natives, Generation Y and Generation Z, will be looked at more closely with regard to PSU, as these generations are each going through different developmental tasks [77,78]. Therefore, we assume that there are also differences between Generation Y and Generation Z in the expression of PSU (H2).

Consistent with previous research [47,61,62,63,64], personality traits and psychopathological symptoms should have an impact on PSU; therefore, we hypothesize that PSU correlates with personality and the presence of psychopathological symptoms (H3). As described by Ahn and Jung and Prensky, digital immigrants and digital natives differ strongly in their usage behavior and their view towards smartphones, and the reasons for the emergence of PSU [73,74]. For this reason, it is hypothesized that PSU will correlate with different personality traits and psychopathological symptoms in digital immigrants (persons over 40 years of age) than in digital natives (persons under 40 years of age) (H4).

In addition, digital natives can be divided into Generations Y and Z. These, in turn, differ significantly in their lifestyles and the development tasks they have to master [76,77], and as Echeburúa and de Corral were able to determine, younger people are more inclined to form PSU [78]. For this reason, the hypothesis is proposed that PSU correlates with different personality traits and psychopathological symptoms in members of Generation Y (persons over 25 years of age) than in members of Generation Z (persons under 25 years of age) (H5).

## 2. Materials and Methods

### 2.1. Participants and Procedure

The study sample consists of a total of 399 subjects (*n* = 399). The study included 312 females (*n* = 312), 86 males (*n* = 86), and 1 person who assigned himself to be “diverse” (*n* = 1). The large difference between the sexes can be explained by the fact that a large proportion of the subjects are psychology students and that they are predominantly female. Nevertheless, it can be seen that the male gender is sufficiently represented with 86 subjects and that the sample distribution of the mean is approximately normally distributed [80,81]. The average age was 25.9 years (*M_age_* = 25.9) with a minimum of 14 years (*Min* = 14) and a maximum of 67 years (*Max* = 67). In this regard, the sample had a standard deviation of 11.1 (*SD_age_* = 11.1). In terms of generations, 44 members of the digital immigrants generation (*n* = 44), 355 members of the digital natives generation (*n* = 355), 35 members of Generation Y (*n* = 35), and 320 members of Generation Z (*n* = 320) participated. Regarding the occupation, a total of 255 students participated in the study (*n* = 256). This was followed by the group of working people with 96 participants (*n* = 96), 44 pupils (*n* = 44), and only one participant without employment (*n* = 1).

Subjects were recruited through the network of the University of Koblenz Landau. To increase the incentive to participate in the survey, 10 EUR 20 Zalando vouchers were raffled among all participants. The entire data collection was carried out online via the platform SoSciSurvey. The survey period was from 11 January 2021 to 24 January 2021. During this period the second German lockdown due to the COVID-19 pandemic also took place from December 2020 to May 2021. The online survey took approximately 15 min to complete and consisted of a questionnaire battery consisting of a sociodemographic questionnaire, the BFI-10 questionnaire [82], the MPPUS [30], followed by the ISR [83]. All questions were asked in German.

### 2.2. Material and Measures

The materials used are standardized questionnaires with all information provided in the self-assessment. First, sociodemographic data (gender, age, state, occupation, and smartphone-usage time) were collected.

### 2.3. MPPUS-27

The Mobile Phone Problematic Use Scale (MPPUS-27) by Bianchi and Phillips is used to investigate problematic smartphone use and consists of 27 items [30]. The German version of the MPPUS used in this study is a self-translation that was tested and created using the back-translation method and can be found in Appendix A [84]. Through the ten-point Likert scale from not at all true (1) to completely true (10), the aspects of tolerance, negative effects on everyday life, escape from problems, loss of control, cravings, and withdrawal in the social, family, and professional context are queried. In addition, there is the question about the time factor, i.e., the duration of smartphone use and how much time is lost through it. The MPPUS was calculated using the sum score from all 27 items and has an excellent internal consistency in this study with a value of Cronbach’s alphas of >0.9 [85].

### 2.4. ISR

The ICD-10 Symptom Rating (ISR) by Tritt et al. is a self-assessment regarding the status and severity of psychopathological symptoms [83]. It is based on the International Statistical Classification of Diseases and Related Health Problems. As such, it serves to standardize and simplify medical diagnoses by defining the symptoms and diagnoses of specific diseases. The ISR consists of five scales, each represented by three to four items and formed using the mean. The scales include depression, anxiety disorders, obsessive-compulsive disorders, somatizations, and eating disorders. The rating uses a five-point scale from strongly disagree (0) to strongly agree (4). The ISR has excellent to acceptable internal consistencies in this study with Cronbach’s alpha values of >0.8 for depression, >0.8 for eating disorders, >0.8 for anxiety disorders, >0.8 for obsessive-compulsive disorders, and >0.8 for somatizations [85].

### 2.5. BFI-10

The BFI-10 [82] measures the Big Five personality traits of extraversion, neuroticism, agreeableness, conscientiousness, and openness. The BFI-10 is based on the BFI with originally 44 items [86], which was shortened to 10 items for economic reasons. The ten items of the inventory consist of two items per dimension, which are represented by one positive and one negative item. The ten questions are answered with the aid of a five-point rating scale ranging from “strongly disagree” (1) to “strongly agree” (5) and the five scales are formed with the use of the mean value. The average time required to complete the inventory is one minute [82]. The BFI-10 has good to unacceptable internal consistencies in this study with Cronbach’s alpha values of >0.6 for neuroticism, >0.5 for conscientiousness, >0.6 for openness, >0.3 for agreeableness, and >0.8 for extraversion [83]. The Cronbach’s alpha values mentioned here should be viewed with caution due to the low number of items per dimension and thus low heterogeneity, which is why Rammstedt et al. prefer retest reliability to internal consistency. In the original study by the authors of the test, this was 0.75 measured over a period of 6 weeks, but could not be carried out here due to a lack of repeated measurements [87].

### 2.6. Statistical Analysis

Data analysis for this study was performed using IBM SPSS version 27 statistical software. The data analysis consists of descriptive statistics (means and standard deviations) and inferential statistical analysis procedures, calculation of mean differences between generations, primarily multiple linear regressions on the variables using PSU (measured by the MPPUS) as a criterion, and participants’ psychopathological symptoms (measured by the ISR) and personality traits (measured by the BFI-10) as predictors (a) for the entire sample and (b) moderation analysis for testing different effects for digital natives and digital immigrants and (c) moderation analysis for testing different effects for Generation Y and Generation Z.

## 3. Results

In Table 1, the bivariate correlations between all variables used can be seen for the entire data set.

Table 2 shows the mean values and standard deviations of the raw scores for the MPPUS and the subscales for the ISR and the BFI-10 for the entire sample and broken down by the subgroups of digital immigrants and digital natives divided into Generations Y and Z.

The conclusion about differences (H1 and H2) are drawn from t-tests. The results confirm that there are statistically significant differences between digital immigrants and digital natives in the expression of PSU (H1) *t*(398) = 7.854, *p* < 0.001, *d* = 1.381, the personality traits neuroticism *t*(397) = 3.201, *p* = 0.001, *d* = 0.563 and conscientiousness *t*(397) = −3.152, *p* = 0.002, *d* = 0.554, as well as in the psychopathological symptoms of depression *t*(396) = 4.789 *p* < 0.001, *d* = 0.853, anxiety *t*(396) = 2.882, *p* = 0.004, *d* = 0.513, and compulsivity *t*(396) = 2.527, *p* = 0.012, *d* = 0.450. Concerning Generations Y and Z (H2), significant mean differences were only found in the expression of the PSU *t*(353) = 2.619, *p* = 0.009, *d* = 0.461.

Afterward, we calculated a multiple linear regression analysis using the inclusion method. Regression was first performed on the entire data set (H3). Then, moderation analyses were run to see if there were different effects of predictors according to digital natives and digital immigrants (H4). The same procedure was then performed for Generations Y and Z (H5). In all regressions, gender was included in the analysis as a control variable.

The prerequisites for the analysis were checked and fulfilled.

For H3, one subject was excluded from the calculation as an outlier. Regression for H3 revealed that psychopathological symptoms for obsessive-compulsive disorders, depression, the personality trait conscientiousness, and gender were significant predictors of PSU (Table 3). The psychopathological symptoms for eating disorders, anxiety, and somatization, as well as the personality traits extraversion, neuroticism, openness, and agreeableness, could not explain any incremental variance and were therefore not included further.

The four significant predictors explained 30% of the variance in PSU (F(11,385) = 15.264, *p* < 0.001, *R*^2^ = 0.304), which corresponds to a high variance explanation (ibid.).

For H4, moderation analysis was calculated for the two generations of digital immigrants (individuals over 40 years of age) and digital natives (individuals under 40 years of age). One subject from digital natives was excluded from the calculation as an outlier. The moderation analysis was run to determine whether the interaction between generation and psychopathological symptoms and personality traits predicts PSU. Results show that the generation moderated the effect between eating disorder symptoms and PSU significantly, F(13, 383) = 17.452, *p* = 0.006, Δ*R*^2^ = 0.013) predicting 1.3% of the variance, as seen in Table 4.

**Figure 1:**
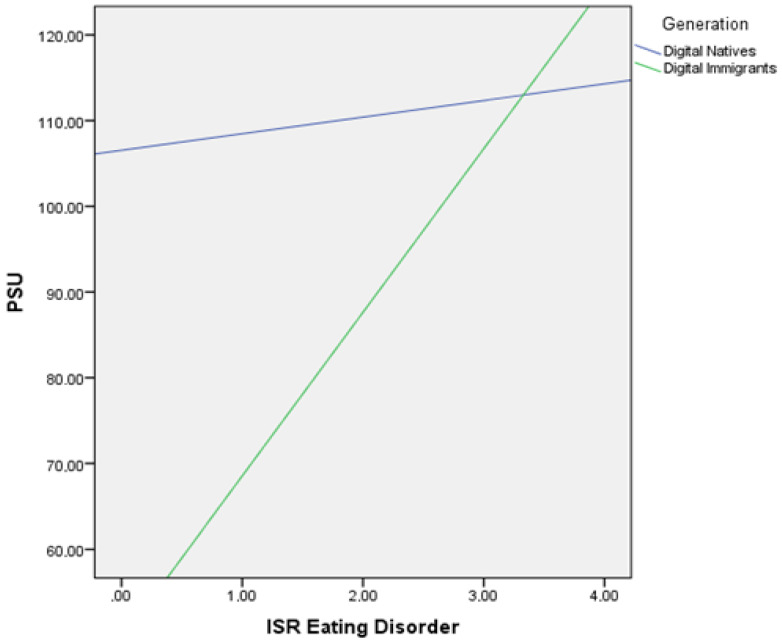
Interaction plot between eating disorder symptoms and PSU for digital natives and digital immigrants.

Figure 1 shows the interaction plot of the relationship between eating disorder symptoms and PSU by generation.

To answer H5, moderation analysis was again calculated for the two Generations Y (persons over 25 years) and Z (persons under 25 years). One subject from Generation Z was excluded from the calculation as an outlier. The moderation analysis was run to determine whether the interaction between generation and psychopathological symptoms and personality traits predicts PSU. Results show that the generation moderated the effect between conscientiousness (F(13, 339) = 11.521, *p* = 0.001, Δ*R*^2^ = 0.022) as well as depression symptoms (F(13, 339) = 10.834, *p* = 0.035, Δ*R*^2^ = 0.009) and PSU significantly, predicting 2.2% of the variance for conscientiousness and 0.9% for depression symptoms, which corresponds to a small variance explanation for conscientiousness (ibid.), as seen in Table 5. 

**Figure 2:**
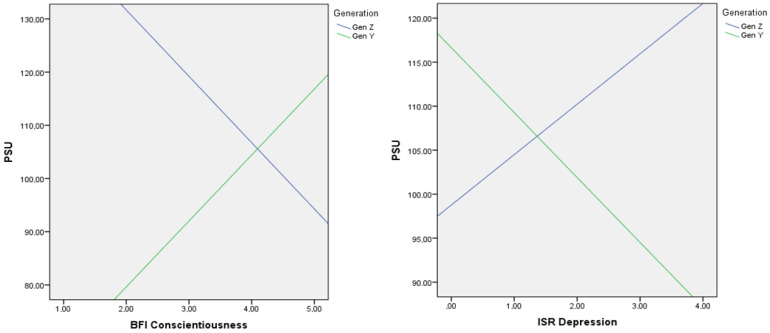
Interaction plot between conscientiousness and PSU and depression and PSU for Generation Y and Generation Z.

Figure 2 shows the interaction plot of the relationship between conscientiousness as well as depression symptoms and PSU by generation.

## 4. Discussion

The present study dealt with the psychopathological and personality-related predictors of problematic smartphone use in different generational groups. 

The results of H1 show differences between digital immigrants and digital natives in the expression of PSU, neuroticism, conscientiousness, depression, anxiety, and compulsivity. Concerning H2, for Generations Y and Z, differences were only found in the expression of the PSU. Regression for H3 revealed that psychopathological symptoms for obsessive-compulsive disorders and depression, the personality trait conscientiousness, and gender were significant predictors of PSU. Moderations have shown that being a digital immigrant or digital native moderates the impact of eating disorders on PSU (H4). Belonging to Generation Y or Generation Z moderates the influence of conscientiousness and depression on PSU (H5). Thus, it shows that in different generations, different factors seem to play a role in the development and maintenance of PSU. 

These results are consistent with existing research. First, the differences in the generations (H1 and H2) can be explained by the fact that individuals are in different stages of life and thus cope with different developmental tasks [76,77].

Regarding the results of H3, correlates and the effect as a predictor of depression or depressive symptoms as well as symptoms of OCD have been demonstrated several times in research [15,39,40,47,48,49]. In this context, smartphones are used as a dysfunctional coping strategy to distract oneself from negative emotions and escape from this affective state [28,87,88]. In terms of personality factors, low conscientiousness is associated with lower achievement orientation and poorer organizational skills. Accordingly, it is easier for them to lose themselves in the endless possibilities of the smartphone and to tend toward PSU [89,90]. 

Looking at the moderation, it becomes apparent that the generational groups moderate the effect of psychopathological symptoms of eating disorders on PSU. While PSU is consistently elevated for digital natives, digital immigrants are shown to be more prone to PSU when they have more severe eating disorder symptoms. Since this is a new finding, we can only speculate about the reasons for this. One possible reason could be that digital immigrants with eating disorder symptoms are more likely to withdraw and, in line with the Pathway Model, more likely to use the smartphone [14].

Looking at the moderation, it becomes apparent that the generational groups moderate the effect of psychopathological symptoms of depression on PSU. While Generation Z individuals with depressive symptoms tend to have more severe PSU, the reverse is true for Generation Y individuals. Persons of this generation with depressive symptoms tend to have lower PSU. One possible reason for this could be that Generation Y tends to withdraw, while Generation Z, in line with the Pathway Model, tends to seek reassurance from their smartphones [14].

Another moderation result is that generational groups are found to moderate the effect of the personality trait conscientiousness on PSU. While conscientious individuals of Generation Z tend to be less inclined to PSU, the effect for Generation y is exactly the opposite. The more conscientious someone is, the stronger PSU is. One possible reason for this could be that people in Generation Z are still at school or studying and the smartphone is more of a nuisance, which is why they have to discipline themselves not to use it. Generation Y, on the other hand, is usually in the middle of their working lives and has both professional and private obligations, which is why conscientious people always have their smartphone with them and use it as much as possible in order to be able to react to every obligation.

An important aspect to be considered is the confounding of the generation with the developmental phase and thus the problem of cross-sectional bias. Certain psychological symptoms occur more frequently in adolescence than in adulthood (see the tests conducted for mean differences, particularly between digital natives and digital immigrants). Accordingly, it is possible that the different relevance and expression depending on the generation also has an impact on what influences the PSU. In that sense, it could be assumed that if a disorder occurs less frequently in one generation per se, it differentiates better there and is thus a better predictor.

### Limitations

The study suffers from a few limitations. One is that the survey was self-reported, which could lead to a biased picture. In addition, the sample sizes of the subgroups differ greatly; the Generation Y group, in particular, is very small and does not fulfill the statistical power, which means that a generalization of the results is only possible to a limited extent. Thus, future studies should aim for groups of equal size. In addition, it should be noted that only slightly less than a quarter of the subjects were male, which means that the results can be used primarily to describe the factors influencing PSU in women. Moreover, the SARS-CoV pandemic can be expected to exacerbate the increase in psychopathological symptoms and PSU as social interactions shift from the analog to the digital world, which means that outcomes must always be viewed in light of the exceptional temporal situation. In the future, it will be important to conduct prospective longitudinal studies on the topic in order to be able to show representative results with causal directions and additionally, as described before, to better control the confounding between the respective development phases and the generation.

## 5. Conclusions

The present study should first contribute to investigating different generation-specific subgroups to identify PSU predictors specifically. It could be shown that in different generations, different predictors play an important role, which indicates a relationship between developmental phenomena and clinical manifestations and are also evidence of the interplay of age-related (adolescence vs. early adulthood) and epochal-historical developmental influences (degree of digitalization of society) [91]. 

The topicality of the issue was highlighted not least by the recent Facebook scandals, through which it became known that the Facebook corporation knew and willingly accepted the negative effects on young people’s mental health [92]. Given this information to work on the specific predictors according to the respective life stages and developmental tasks to be completed, the smartphone can be a healthy enrichment and simplification of daily life without ending in excessive use or problematic dependence.

## Figures and Tables

**Table 1:** Bivariate correlations after Pearson with ISR, BFI-10, and MPPUS (total sample).

	1	2	3	4	5	6	7	8	9	10
BFI										
Extraversion										
Neuroticism	−0.222 **									
Openness	−0.011	−0.038								
Conscientiousness	0.044	−0.085	0.092							
Agreeableness	0.091	−0.057	0.016	0.147 **						
MPPUS	−0.015	0.267 **	0.088	−0.340 **	−0.078					
Psychopathological Symptoms										
Depression	−0.154 **	0.378 **	0.040	−0.274 **	−0.156 **	0.410 **				
Anxiety	0.303 **	0.532 **	0.043	−0.212 **	−0.126 *	0.341 **	0.508 **			
Obsessive-Compulsive	−0.120 *	0.388 **	0.052	−0.128 *	−0.029	0.327 **	0.446 **	0.561 **		
Somatization	−0.137 **	0.331 **	0.072	−0.073	−0.150 **	0.297 **	0.397 **	0.506 **	0.392 **	
Eating Disorder	0.015	0.159 **	0.066	0.026	−0.053	0.233 **	0.304 **	0.247 **	0.239 **	265 **

* indicates *p* < 0.05, ** indicates *p* < 0.01.

**Table 2:** Descriptive statistics for ISR, BFI-10, and MPPUS (total sample, digital immigrants, digital natives, Generation Y, and Generation Z).

	Total Sample (*n* = 399)	Digital Immi-grants (*n* = 44)	Digital Natives (*n* = 355)	Generation Y (*n* = 35)	Generation Z (*n* = 320)
	Mean (SD)				
Personality					
Neuroticism	3.04 (0.97)	2.60 (1.06)	3.10 (0.95)	2.96 (0.92)	3.11 (0.95)
Agreeableness	3.33 (0.78)	3.33 (0.82)	3.34 (0.78)	3.27 (0.68)	3.34 (0.79)
Conscientiousness	3.53 (0.82)	3.90 (0.66)	3.49 (0.83)	3.54 (0.69)	3.48 (0.85)
Openness	3.65 (0.95)	3.48 (0.91)	3.67 (0.95)	3.71 (0.98)	3.66 (0.95)
Extraversion	3.47 (0.95)	3.50 (0.82)	3.47 (0.97)	3.57 (0.98)	3.45 (0.97)
Psychopathological Symptoms					
Depression	2.42 (0.94)	1.80 (0.87)	2.5 (0.92)	2.41 (0.86)	2.51 (0.93)
Obsessive-Compulsive	1.86 (0.92)	1.53 (0.85)	1.9 (0.92)	1.89 (0.99)	1.91 (0.92)
Anxiety	2.00 (0.94)	1.62 (0.78)	2.05 (0.95)	1.96 (1.00)	2.06 (0.94)
Eating Disorder	1.89 (1.03)	1.66 (0.75)	1.92 (1.05)	1.87 (0.98)	1.92 (1.06)
Somatization	1.42 (0.79)	1.29 (0.54)	1.44 (0.81)	1.42 (0.83)	1.44 (0.81)
PSU					
MPPUS	107.04 (36.34)	69.23 (29.18)	111.63 (34.39)	96.31 (29.80)	113.31 (34.48)

**Table 3:** Predictors of PSU.

	Total Sample (*n* = 398)				
	ß	*t*	*p*	*R* ^2^	*Adjusted R* ^2^
Personality					
Neuroticism	0.081	1.560	0.120	0.304	0.284
Agreeableness	−0.006	−0.141	0.888		
Conscientiousness	−0.282	−6.170	<0.001		
Openness	0.078	1.791	0.074		
Extraversion	0.062	1.371	0.171		
Psychopathological Symptoms					
Depression	0.166	3.063	0.002		
Obsessive-Compulsive	0.122	2.243	0.025		
Anxiety	0.009	0.142	0.887		
Eating Disorder	0.091	1.960	0.051		
Somatization	0.101	1.963	0.050		
Gender	−0.113	−2.472	0.014		

**Table 4:** Moderation for digital natives and digital immigrants.

	Digital Natives (*n* = 354)/Digital Immigrants (*n* = 44)			
	ß	*t*	*p*	Δ*R*^2^
*Personality*				
Generation*Neuroticism	0.081	0.715	0.475	0.001
Generation*Agreeableness	0.153	0.867	0.386	0.001
Generation*Conscientiousness	0.046	0.186	0.852	<0.001
Generation*Openness	−0.214	−1.301	0.194	0.003
Generation*Extraversion	−0.141	−0.772	0.441	0.001
*Psychopathological Symptoms*				
Generation*Depression	−0.012	−0.121	0.904	<0.001
Generation*Obsessive-Compulsive	−0.072	−0.832	0.406	0.001
Generation*Anxiety	−0.101	−1053	0.293	0.002
Generation*Eating Disorder	0.272	2.762	0.006	0.013
Generation*Somatization	−0.048	−0.448	0.655	<0.001

* indicates the interaction between variables.

**Table 5:** Moderation for Generation Y and Generation Z.

	Generation Y (*n* = 319)/Generation Z (*n* = 35)			
	ß	*t*	*p*	Δ*R*^2^
*Personality*				
Generation*Neuroticism	−0.200	−1.261	0.208	0.003
Generation*Agreeableness	0.180	0.786	0.432	0.001
Generation*Conscientiousness	0.783	3.300	0.001	0.022
Generation*Openness	−0.131	−0.696	0.487	0.001
Generation*Extraversion	−0.034	−0.189	0.850	<0.001
*Psychopathological Symptoms*				
Generation*Depression	−0.294	−2.122	0.035	0.009
Generation*Obsessive-Compulsive	−0.055	−0.533	0.594	0.001
Generation*Anxiety	−0.111	−1.051	0.294	0.002
Generation*Eating Disorder	−0.077	−0.754	0.452	0.001
Generation*Somatization	−0.035	−0.374	0.709	<0.001

* indicates the interaction between variables.

## Data Availability

The data used to analyze the results reported here are available for public review at https://mfr.osf.io/render?url=https%3A%2F%2Fosf.io%2F8s9vn%2Fdownload (accessed on 10 December 2021).

## Appendix A

German version of the MPPUS created using the back-translation method

1. Ich kann nie genug Zeit mit meinem Smartphone verbringen.
2. Ich habe mein Smartphone benutzt, um mich besser zu fühlen, wenn ich mich schlecht fühlte.
3. Ich finde mich in Situationen wieder, in denen ich von meinem Smartphone eingenommen bin, obwohl ich andere Dinge tun sollte und das verursacht Probleme.
4. Alle meine Freunde besitzen ein Smartphone.
5. Ich habe versucht vor anderen zu verbergen, wie viel Zeit ich mit meinem Smartphone verbringe.
6. Ich schlafe weniger aufgrund der Zeit, die ich mit meinem Smartphone verbringe.
7. Ich habe Smartphonerechnungen erhalten, die ich mir nicht leisten konnte.
8. Wenn ich für einige Zeit keinen Empfang auf meinem Smartphone habe, beschäftigen mich die Gedanken, einen Anruf oder eine Nachricht zu verpassen.
9. Manchmal, wenn ich mit dem Smartphone telefoniere und dabei andere Dinge tue, werde ich von der Unterhaltung mitgerissen und achte nicht darauf was ich gerade eigentlich tue.
10. Die Zeit, die ich mit meinem Smartphone verbringe, hat in den letzten 12 Monaten zugenommen.
11. Ich habe mein Smartphone benutzt, um mit anderen zu sprechen, als ich mich isoliert fühlte.
12. Ich habe versucht, weniger Zeit mit meinem Smartphone zu verbringen, aber es gelingt mir nicht.
13. Ich finde es schwierig, mein Smartphone auszuschalten.
14. Ich bin besorgt, wenn ich einige Zeit nicht meine Nachrichten kontrollieren kann oder mein Smartphone nicht eingeschaltet habe.
15. Ich träume häufig von meinem Smartphone.
16. Meine Freunde und Familie beschweren sich über meine Smartphonenutzung.
17. Wenn ich kein Smartphone hätte, wäre es für meine Freunde schwierig, mit mir in Kontakt zu treten.
18. Meine Produktivität ist als direkte Folge der Zeit, die ich mit dem Smartphone verbringe, gesunken.
19. Ich habe Wehwehchen, die mit der Benutzung meines Smartphones zusammenhängen.
20. Ich bin für längere Zeiträume als vorgesehen mit dem Smartphone beschäftigt.
21. Es gibt Zeiten, in denen ich lieber das Smartphone benutze, als mich mit anderen dringenderen Dingen zu befassen.
22. Ich komme oft zu spät zu Terminen, weil ich mit dem Smartphone beschäftigt bin, obwohl ich es nicht sollte.
23. Ich werde gereizt, wenn ich mein Smartphone für Besprechungen, beim Abendessen oder im Kino ausschalten muss.
24. Mir wurde gesagt, dass ich zu viel Zeit mit meinem Smartphone verbringe.
25. Mehr als einmal war ich in Schwierigkeiten, weil mein Mobiltelefon während einer Besprechung, eines Vortrags oder im Theater ausgeschaltet war.
26. Meine Freunde mögen es nicht, wenn mein Smartphone ausgeschaltet ist.
27. Ich fühle mich ohne mein Smartphone verloren.
